# Drilling technique of the internal auditory canal in vestibular schwannoma surgery assisted by neuronavigated autofocus microscope

**DOI:** 10.1007/s00701-025-06679-1

**Published:** 2025-09-27

**Authors:** Maddalena Dardo, Luca Campagnaro, Andrea Boschi, Serena Tola, Franco Trabalzini, Alessandro Della Puppa

**Affiliations:** 1 Department of Neuroscience, Psychology, Pharmacology and Child Health (NEUROFARBA), University of Florence, Florence, Italy; 2 Department of Neurosurgery, Careggi University Hospital, Florence, Italy; 3 Meyer IRCCS Children’s Hospital Florence, Florence, Italy

**Keywords:** Neuronavigated autofocus microscope, Drilling of the internal auditory canal, Heads-up display microscope, Vestibular schwannomas, Retrosigmoid approach

## Abstract

**Background:**

The wide anatomical variability of temporal bone structures makes the application of neuronavigation particularly useful. This technical note outlines an IAC drilling technique performed with the assistance of a neuronavigated autofocus microscope to enhance intraoperative anatomical orientation, facilitate tailored bone removal and prevent injuries at the intraosseous petrous structures.

**Method:**

From January 2023 to January 2024, twenty-one out of thirty-six patients with vestibular schwannoma underwent a retrosigmoid transmeatal approach with IAC drilling assisted by the neuronavigated autofocus microscope. The technique employed the autofocus function of the surgical microscope as an active navigation pointer, with real-time trajectory feedback display through the heads-up display. This setup enables continuous intraoperative adaptation of the drilling path to individual anatomical landmarks.

**Results:**

Postoperative high-resolution CT imaging confirmed preservation of critical intraosseous structures in all but one case, which showed limited endolymphatic duct violation. No injuries to the posterior semicircular canal, common crus, or jugular bulb were observed. Complete tumour resection was achieved in all patients. The technique has enabled different drilling angles and trajectories tailored to individual patient anatomy.

**Conclusions:**

The IAC drilling, performed under the assistance of a neuronavigation-integrated autofocus microscope, provides a tailored anatomy-guided approach. This technique facilitates individualized exposure of the intrameatal tumour component while supporting the preservation of critical intraosseous petrous structures. By continuously adapting the drilling trajectory to the patient’s specific anatomy, it enables a controlled removal of the IAC posterior wall and may contribute to reducing the risk of unintended structural injury.

**Supplementary Information:**

The online version contains supplementary material available at 10.1007/s00701-025-06679-1.

## Introduction

During vestibular schwannoma surgery via the retrosigmoid approach, the removal of the posterior wall of the internal auditory canal (IAC) is essential to expose the portion of the tumour lying within the canal, and to achieve complete tumour resection [5–7, 15–17, 20, 21]. The goal of vestibular schwannoma (VS) surgery should be total resection of the tumour and preservation of neurological functions such as hearing and facial nerve integrity [16]. In order to preserve hearing, it is essential to preserve the anatomical integrity of the cochlear nerve and the labyrinthine structures [3, 4] (endolymphatic duct and sac) which, together with the jugular bulb, are put at risk during the drilling of the posterior wall of the IAC, due to their anatomic variability and the lack of intraoperative landmarks indicating their precise location in the petrous bone [13, 18]. These factors have limited the identification of a standard angle and extension of drilling during the procedure, highlighting the importance of high-resolution computed tomography (HR-CT) in the preoperative planning and image-guided drilling tailored to anatomical individuality [8, 9, 11, 15].

This technical note describes our drilling technique of the IAC assisted by a real-time neuronavigated autofocus microscope (using merged pre-operative HR-CT and MRI). We examine drawbacks and nuances of this technique to prevent labyrinth and JB damages, and to facilitate a tailored removal of the IAC posterior wall based on patient variability.

## Methods

From January 2023 to January 2024, twenty-one out of the thirty-six patients operated on for unilateral vestibular schwannoma at our Institution underwent surgery via retrosigmoid transmeatal approach using the drilling technique of the IAC guided by neuronavigated autofocus microscope.

In our cohort, preoperative HR-CT images and MRI were merged on the navigation system (Medtronic Stealth 8) and directly visualized by the lead surgeon on the microscope (Zeiss, KINEVO 900) as an overlay in the upper right quarter of the surgical field. The focal length of the microscope served as the localization probe and was set in autofocus mode.

The drilling was planned intraoperatively by the primary surgeon, who identified the safe drilling corridor on the petrous bone using CT-MRI data displayed on the microscope. The drilling angle and trajectory were customized based on the patient's anatomical peculiarities. Finally, the drilling distances and intraosseous petrous structures’ integrity were evaluated on postoperative HR-CT.

A retrospective review of intraoperative technical notes and data was performed. We focused our evaluation on the postoperative morphological integrity of the intraosseous petrous structures on postoperative HR-CT, and on the usefulness of navigation in performing patient-tailored drilling of the IAC. We describe our drilling technique of the IAC and discuss the drawbacks and nuances.

### Case series and preoperative planning

Demographic data, tumour grade classification, tumour size, and petrous bone peculiarities of the patient study group are summarized in Table 1.

**Table 1:** Patients’ and tumours’ characteristics.

Patient Number	Age (years), Sex	Tumor Grade (Koos, Hannover)	Tumor classification according to the relationship between the LSL and the labyrinth: lateral type (L), on-the-line (O), and medial type (M)	Cisternal Tumor Size (mm)	Intracanal Tumor Extension (%)	HJB (Y/N)	Pneumatized petrous apex (Y/N)
1	33, F	3, T3b	L	20	73	N	Y
2	61, F	3, T3b	M	20	52	N	N
3	49, F	3, T3b	M	27	69	N	N
4	39, M	3, T3b	L	19	59	N	N
5	53, F	3, T2	O	5	76	N	N
6	64, F	3, T3b	L	18	100	N	Y
7	71, F	4, T4b	L	30	73	N	Y
8	63, F	2, T3a	M	11	100	N	N
9	56, F	2, T3a	L	10	44	N	N
10	49, F	3, T3b	L	28	100	N	N
11	75, M	4, T4a	O	31	89	N	N
12	61, F	4, T4b	M	52	44	N	N
13	51, M	1, T1	L	Intracanalicular	87	N	Y
14	47, F	4, T4b	O	51	81	N	N
15	67, F	3, T3b	O	25	77	N	Y
16	56, M	4, T4b	L	3	39	N	Y
17	51, F	3, T3b	O	29	100	N	N
18	66, F	4, T4b	L	40	100	Y	N
19	49, M	4, T4a	L	36	100	N	N
20	49, F	4, T4b	L	45	96	N	N
21	72, M	3, T3a	O	25	100	N	Y

Tumor grade was defined according to Koos and Hannover classifications. The line between the outermost edge of the tumour and the medial side of the sigmoid sinus was defined as the lateral safe line (LSL). According to the relationship between the LSL and the labyrinth, the tumours were classified as lateral type (L), on-the-line (O), and medial type (M) tumours. The intracanal tumour extension was calculated as a percentage of tumour extension along the longitudinal axis of the IAC

All patients underwent preoperative HR-CT scans and gadolinium-enhanced MRI. HR-CT bone-window, T1 Gd, and FIESTA imaging were merged in DICOM format on the navigation system and directly displayed on the upper right quarter of the microscope view during the procedure. The laser of the microscope and intraoperative neuronavigation were synchronized.

The neuronavigated registration procedure was performed on HR-CT scans. The following accuracy criteria were adopted: a mean registration error of less than or equal to 1.5 mm, and a mean registration error within the surgical area of less than 1 mm. The latter was considered to correspond to the high-accuracy green area, automatically defined by the neuronavigation system during the registration procedure.

### Surgical technique

Patients underwent the surgical procedure in the park bench position, with the head fixed in a three-pin head holder. A retroauricolar linear skin incision was made behind the mastoid body, and a standard retrosigmoid craniotomy was tailored using the navigation system. The dura was opened in a C-shape fashion just inferior to the transverse and sigmoid sinuses, and dural flap was reflected medially. The cerebellomedullary cistern was opened to drain cerebrospinal fluid and relax cerebellum. This manoeuvre allowed exposure of the tumour and the posterior wall of the IAC with minimal cerebellar dynamic retraction. In vestibular schwannoma Koos grade III-IV, initial debulking of the extrameatal portion of the tumour was performed, in addition to the usual opening of the cerebellopontine cistern before approaching the internal auditory canal.

Neuronavigation-merged HR-CT and MRI datasets, displayed on the microscope, allowed the primary surgeon to identify intraosseous petrous structures and intrameatal tumor extension in real time, by adjusting the focus according to the microsurgical view and the trajectory toward the IAC. Special attention was paid on identifying the posterior semicircular canal, endolymphatic duct and sac, common crus, and jugular bulb. This technique allowed to plan the drilling angle and the depth of posterior IAC wall removal.

**Fig. 1:**
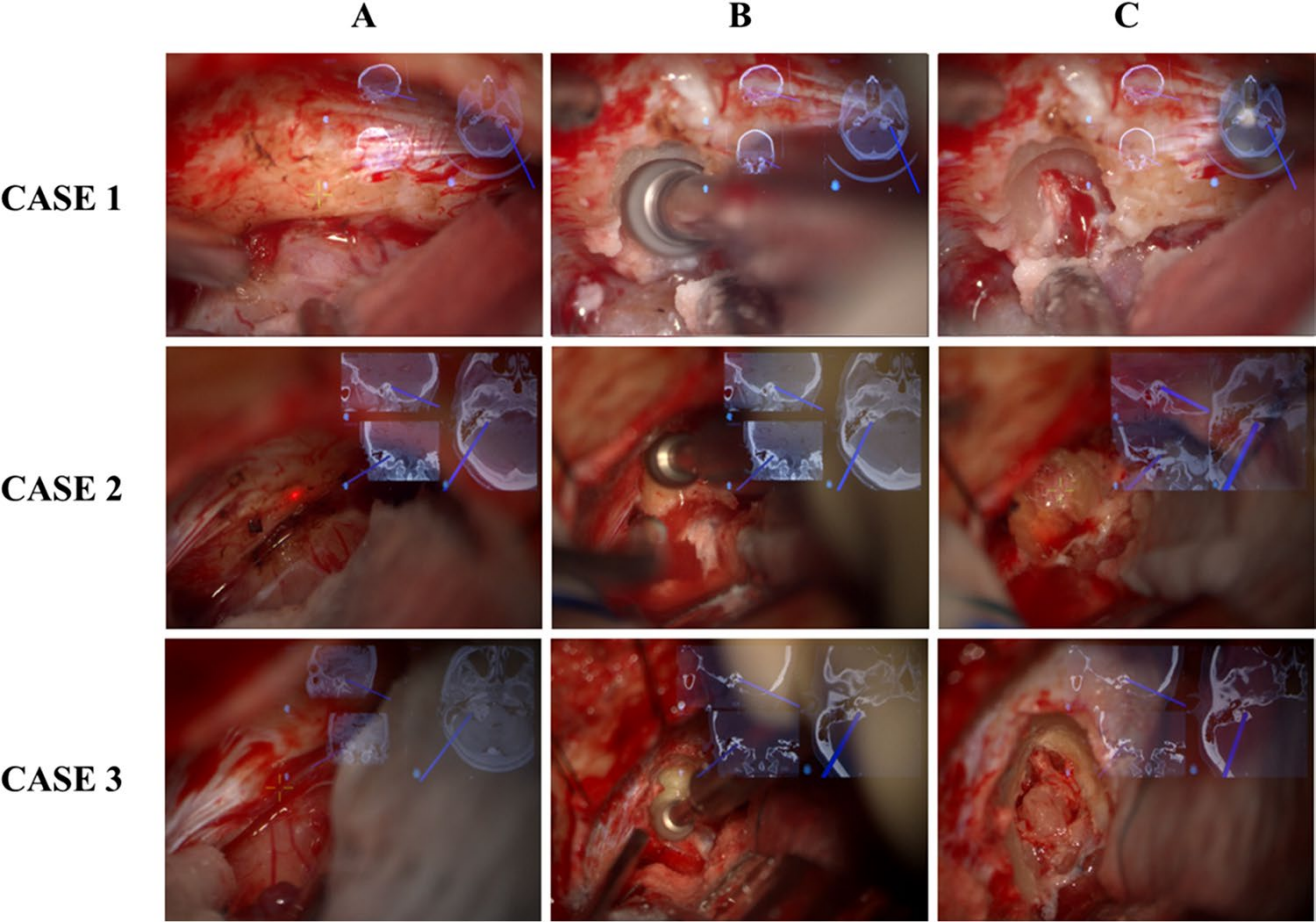
Three cases of our cohort. Microscope images showing the described IAC neuronavigated drilling technique. **A** Identification of intraosseous petrous structures and intrameatal tumour extension to plan a safe drilling corridor. **B** Adjustment of microscope focus during drilling to maintain consistency between neuronavigation imaging and the actual bone plane. **C** Final check of the IAC exposure and distances between the safe drilling corridor and labyrinthine structures

During the drilling procedure, repeated manual adjustments of the microscope focus ensured navigation consistency with the drilled bone plane. The lead surgeon's preference and each surgical step dictated the modulation of the CT-MRI blend, favouring one dataset over the other (Fig. 1).

The rest of the surgery followed standard vestibular schwannoma microsurgical removal principles, accompanied by continuous electrophysiological monitoring and facial nerve mapping.

In order to show our IAC drilling technique in vestibular schwannoma surgery assisted by a neuronavigated autofocus microscope in more details, we made an illustrative video (Video 1, supplementary file).

## Results

Drilling of the IAC assisted by neuronavigated autofocus microscope was performed in twenty-one patients (6 males, 15 females; mean age 56 years ± 21 years; standard deviation [SD], 11 years; range 33 years—75 years). The mean registration error in our case series was 1.4 mm (range 1.1 mm – 1.5 mm; standard deviation [SD], 0.1 mm). In all patients, the mean registration error within the surgical area was less than 1 mm.

High variability in tumour size and intracanalicular extension was observed in our cohort (Table 1). According to the Koos et al. [10] classification, one tumour was grade I, three were grade II, nine were grade III, eight were grade IV. Adopting the Tatagiba [22] and the Yokoyama [21] classification, based on the definition of the lateral safe line (LSL) and the respective labyrinthine structures, we divided our cohort into 4 medial type tumours, 6 on-the-line type tumours, and 11 lateral type tumours. This indicated a higher risk of labyrinth injury during the drilling procedure in four patients. One patient had a high JB, with potential risk of intraoperative haemorrhage and difficult exposure of the IAC. Seven patients had a pneumatized petrous apex, indicating increased risk of postoperative CSF leakage.

We analysed intraoperative IAC drilling, tailored to tumour extension and individual petrous anatomy. The postoperatively measured mean drilling angle at the posterior petrous wall was 38.5° ± 14° (standard deviation [SD], 8.1°; range 25.5° – 53.5°). By measuring the pre- and postoperative length of the IAC, the mean percentage of the posterior wall of the IAC drilled was 66.5% ± 36.5% (standard deviation [SD], 19.8%; range 17%—90%).

**Fig. 2:**
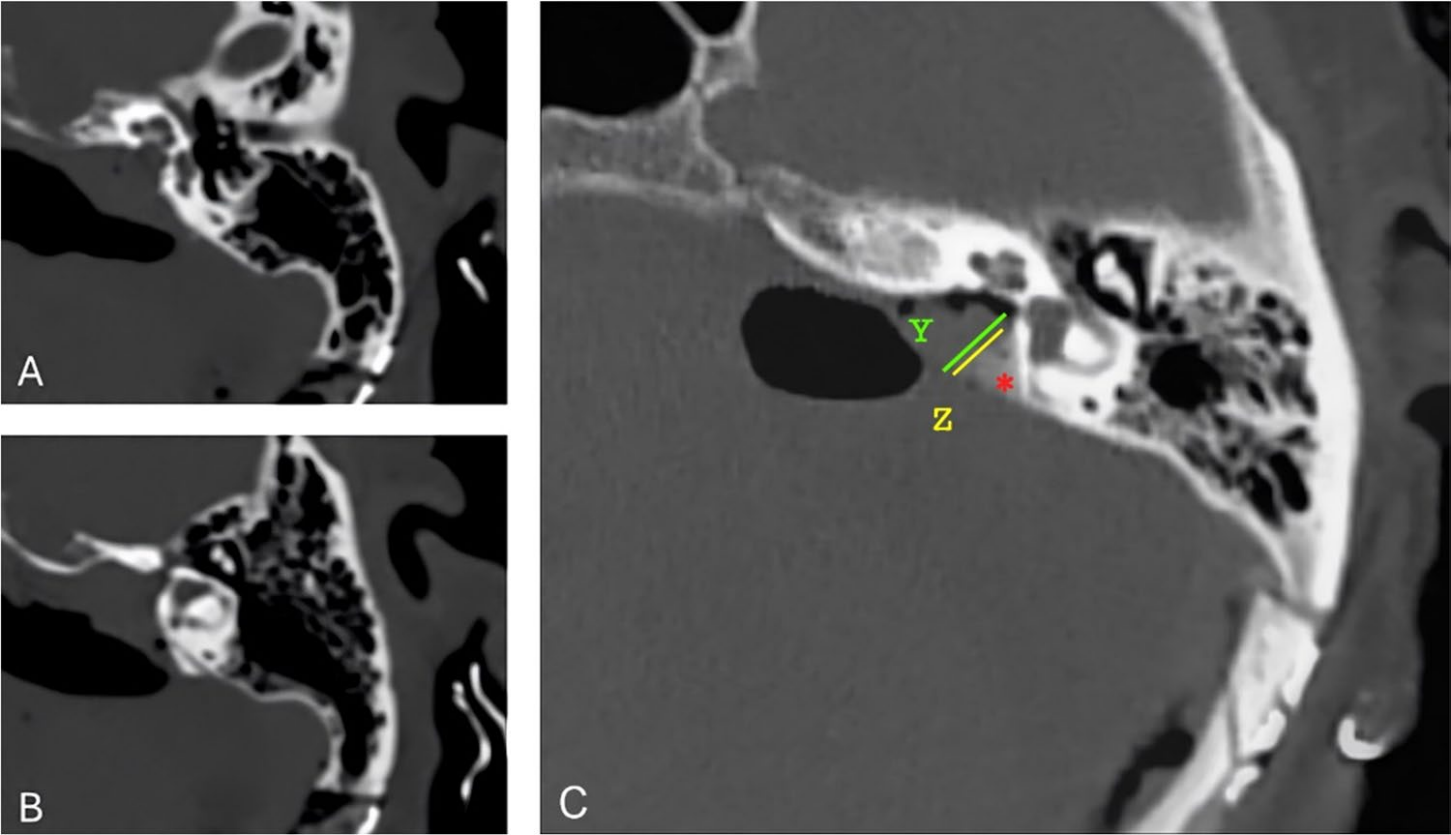
High-resolution CT scan of a patient included in our cohort. **A**,** B** Bone-window postoperative HR-CT images used to assess labyrinthine structural integrity. **C** Pre- and postoperative bone-window HR-CT images merged to measure IAC drilling parameters. Line Z indicates the length of the internal auditory canal drilled. Line Y indicates the total length of the internal auditory canal. The angle of safe neuronavigated drilling is marked with the asterisk

The analysis of postoperative HR-CT images showed that only one patient had structural damage, specifically affecting the endolymphatic duct. All other patients showed structural integrity of the posterior semicircular canal, common crus, endolymphatic duct, and jugular bulb (Fig. 2). Total excision was achieved in all patients.

Drilling of the IAC assisted by real-time neuronavigation-guided microscope, was performed without any complications related to this technique in all cases (Table 2).

**Table 2:** Results of the technique

Patient Number	Posterior wall of the IAC drilled (% drilled)	Angle of Safe Drilling (degrees)	Complications (labyrinth and JB integrity)
1	43	45.2	None
2	80	25.5	None
3	90	34.8	Endolymphatic duct violation
4	71	46.7	None
5	75	49.3	None
6	61	35.2	None
7	50	29.7	None
8	74	41.4	None
9	65	43.8	None
10	84	46.0	None
11	75	46.0	None
12	67	53.5	None
13	54	43.2	None
14	69	35.0	None
15	61	30.0	None
16	17	39.8	None
17	81	26.4	None
18	24	26.0	None
19	87	37.8	None
20	88	31.7	None
21	81	41.3	None

## Discussion

In vestibular schwannoma surgery via the retrosigmoid approach, one crucial step in preserving hearing function and achieving complete tumour resection, is the drilling of the posterior wall of the IAC. Preoperative HR-CT is a necessary planning tool [7, 11, 21] given the significant physiological interindividual variability of the temporal bone anatomy, tumour-induced erosion of the IAC, and the intraoperative absence of reliable anatomical landmarks that identify the precise location of intraosseous petrous structures [6]. MRI allows better visualization of the intrameatal tumour extension, providing important information on how deep drilling must be extended into the canal. Therefore, navigation using HR-CT and MRI provides a roadmap for a safe IAC drilling procedure.

Frameless navigation is widely recognized and adopted in skull base surgery, but its use is not yet standardized in temporal bone drilling procedures. In Table 3 we summarized the different neuronavigation techniques for IAC drilling described in literature.

**Table 3:** Previous studies on navigation techniques adopted for IAC drilling in VS surgery via the retrosigmoid transmeatal approach

Authors (Published by, Year) Study Type	Navigation Technique for IAC Drilling
Samii et al. (Neurosurgery, 2000) [17] Cadaveric, radiological, and preliminary clinical study	Microscope-based navigation system using a laser-assisted autofocus microscope for IAC drilling
Pillai et al. (Neurosurgery, 2009) [15] Cadaveric study	HR-CT optical navigation using a pointer, combined with endoscope-assisted drilling of the posterior IAC wall
Scerrati et al. (World Neurosurgery, 2016) [19] Cadaveric study	HR-CT-based optical navigation with a pointer to expose the IAC fundus
Matsushima et al. (World Neurosurgery, 2017) [12] Cadaveric and clinical study	Application of electromagnetic navigation for temporal bone drilling procedures
Ogiwara et al. (J Neurol Surg B Skull Base, 2018) [14] Clinical study	Real-time neuronavigation with a color-coded 3D model of labyrinthine structures overlaid on the microscope view
Jia et al. (Frontiers in Neurosurgery, 2022) [9] Clinical study	Precise and safe exposure of the intrameatal portion of the tumour using optical navigation (on CT-MRI fused images) combined with endoscopy

### Remarks of our technique

We applied a CT-MRI neuronavigated autofocus microscope to perform the IAC drilling procedure, in a manner similar to the technique described by Samii et al [17]. Our aim was to a safe drilling corridor based on the patient’s individual anatomy, leaving only a thin layer of bone above the labyrinthine structures, in accordance with the information provided by the navigation system.

The analysis of our study cohort data revealed a wide range in drilling depth and angle, confirming that standardized drilling parameters are not applicable to every patient. The awareness of significant case-by-case anatomical variability underscores the importance of preoperative IAC drilling planning using CT-MRI and thereby highlights the value of neuronavigation. Moreover, an increased understanding of each patient’s anatomical peculiarities can guide the surgeon into adopting complementary surgical strategies, such as endoscopic assistance in medial-type schwannomas to achieve complete resection [9], or IAC closure with autologous fat grafting in patient with pneumatized petrous apex to further reduce the risk of CSF leakage [1].

In all cases, the CT-MRI neuronavigated images, displayed directly within the operating microscope via the heads-up display feature, provided a significant intraoperative advantage, enabling a tailored surgical strategy and enhancing the surgeon’s confidence particularly in complex cases, such as IAC drilling in patients with a high-riding jugular bulb.

In only one case in our series (4.8%), we observed postoperative damage to the labyrinthine structures. The incidence of iatrogenic inner ear demage during VS surgery performed without neuronavigation has been reported to be between 21.5% – 34.7%, according to Ben-Shlomo et al [2]. These findings are consistent with those previously reported by Tatagiba et al [22] and Yokoyama et al [21]. Our data suggest that neuronavigation may significantly reduce the risk of inner ear injury during VS surgery.

### Limitation of our technique

We evaluated this technique focusing on the postoperative morphological integrity of the labyrinthine structures rather than on clinical auditory outcomes. This decision was made to avoid confounding factors resulting from other iatrogenic causes of hearing impairment. Furthermore, in our cohort, most patients presented with medium- to large-sized vestibular schwannomas and significant preoperative hearing impairment; therefore, an analysis of postoperative auditory outcomes could not be considered a reliable indicator of the technique’s effectiveness in preserving petrous intraosseous structures.

The accuracy of navigation remains the most significant limitation of our technique, and further statistical validation studies are required to define a more precise confidence interval within which a safe drilling corridor can be reliably identified. Bony-surface registration combined with a pivoting C-arc and intraoperative CT appears to represent a strategy capable of increasing neuronavigation accuracy.

Another issue concerns the perpendicular alignment of the laser autofocus. If this is not guaranteed, it can significantly increase navigation error. The surgeon must be thoroughly familiar with the neuronavigated autofocus microscope and the use of the heads-up display feature; otherwise, the visual clutter may become a limiting factor. As a matter of fact, the endolymphatic duct damage occurred in one of the cases using the neuronavigated autofocus microscope and the head-up display was partially overlapping the microsurgical view.

In experienced hands, the dynamic nature of microscope-guided navigation became an advantage. The surgeon can choose whether to emphasize CT or MRI data in the fusion imaging and can temporarily disable the navigation overlay on the microscope to widen the surgical field of view.

## Conclusion

IAC drilling performed under the guidance of a neuronavigation-integrated autofocus microscope provides a tailored, anatomy-driven approach. This technique may contribute to reduce the risk of unintended structural injury and may help enhance surgical confidence in both experienced and less experienced surgeons.

## Supplementary Information

Below is the link to the electronic supplementary material.

ESM 1 (MP4 53.9 MB)

## Data Availability

Data is provided within the manuscript.

## Abbreviations

IAC: internal auditory canal
VS: Vestibular Schwannoma
CT: computer tomography
HR CT: high resolution computer tomography
MRI: magnetic resonance imaging
JB: jugular bulb
HJB: high jugular bulb
LSL: ateral safe line
CSF: cerebrospinal fluid
T1 Gd: post Gadolinium slices T1 weighted
FIESTA: fast imaging employing steady state acquisition
